# Nuts For Babies Study: protocol for a randomised controlled trial in Australia investigating if the risk of developing peanut and cashew nut allergies during infancy can be reduced by a high peanut and cashew nut maternal diet for the first 6 months of lactation

**DOI:** 10.1136/bmjopen-2025-108137

**Published:** 2025-10-09

**Authors:** Thomas R Sullivan, Vicki McWilliam, Michael O’Sullivan, Merryn Netting, Sharon Perrella, Donna Geddes, Mimi Tang, Dianne E Campbell, Kirsten P Perrett, Debra J Palmer

**Affiliations:** 1 SAHMRI Women and Kids, South Australian Health and Medical Research Institute, Adelaide, South Australia, Australia; 2 School of Public Health, The University of Adelaide, Adelaide, South Australia, Australia; 3 Centre for Food Allergy Research, Murdoch Childrens Research Institute, Parkville, Victoria, Australia; 4 Department of Allergy and Immunology, The Royal Children’s Hospital Melbourne, Parkville, Victoria, Australia; 5 Department of Paediatrics, The University of Melbourne, Melbourne, Victoria, Australia; 6 National Allergy Centre of Excellence, Murdoch Children’s Research Institute, Parkville, Victoria, Australia; 7 Immunology Department, Perth Children’s Hospital, Nedlands, Western Australia, Australia; 8 School of Medicine, The University of Western Australia, Perth, Western Australia, Australia; 9 The Kids Research Institute Australia, Nedlands, Western Australia, Australia; 10 School of Medicine, The University of Adelaide, Adelaide, South Australia, Australia; 11 School of Molecular Sciences, The University of Western Australia, Perth, Western Australia, Australia; 12 Centre for Human Lactation Research and Translation, The University of Western Australia, Perth, Western Australia, Australia; 13 Murdoch Children’s Research Institute, Parkville, Victoria, Australia; 14 Department of Allergy and Immunology, The Royal Children’s Hospital Melbourne, Melbourne, Victoria, Australia; 15 Specialty of Child and Adolescent Health, Sydney Medical School, Faculty of Medicine and Health, The University of Sydney, Sydney, New South Wales, Australia; 16 Department of Allergy and Immunology, The Children’s Hospital at Westmead, Sydney, New South Wales, Australia; 17 Nutrition In Early Life, The Kids Research Institute Australia, Nedlands, Western Australia, Australia

**Keywords:** Immunity, Infant, Randomized Controlled Trial, Mothers, NUTRITION & DIETETICS

## Abstract

**Introduction:**

The predisposition to food allergy development and the induction of allergen-specific immune responses appears to be initiated early in infancy. Early exposure to food allergens, such as peanut and cashew nut, via human milk is likely important in initiating oral tolerance and reducing risk of food allergy development. This trial aims to determine if the risk of developing peanut and cashew nut allergy during infancy can be reduced by a high peanut and cashew nut maternal diet during lactation.

**Methods and analysis:**

This is a multisite, parallel, two-arm (1:1 allocation), single-blinded (outcome assessors, statistical analyst and investigators), randomised controlled trial. Target sample size is 4412 participants (2206 per group). Women (aged 18–50 years) with a singleton pregnancy, who are planning to breastfeed and do not have peanut and/or cashew nut allergies are eligible to participate. After obtaining written informed consent, participants are randomised to either a high peanut and cashew nut diet (at least 60 peanuts and 40 cashew nuts per week) or a low peanut and cashew nut diet (no more than 20 peanuts and 12 cashew nuts per week). Participants are asked to follow their allocated diet from birth to 6 months postnatal. Individual lactation consultant advice and support is provided as required. The study’s primary outcome is food challenge proven IgE-mediated peanut and/or cashew nut allergy during infancy (0–18 months). Key secondary outcomes include infant sensitisation to peanut and/or cashew nut. Analyses will be performed on an intention-to-treat basis according to a prespecified statistical analysis plan.

**Ethics and dissemination:**

Ethical approval has been granted from the Western Australian Child and Adolescent Health Service Human Research Ethics Committee (approval number RGS0000006685). Trial results will be presented at scientific conferences and published in peer-reviewed journals.

**Trial registration number:**

Australian New Zealand Clinical Trials Registry (ACTRN ACTRN12624000134527)

**Strengths and limitations of this study**

- This study is a large randomised controlled trial designed with adequate power to assess the effect of higher maternal peanut and cashew nut consumption during the first 6 months of lactation on infant peanut and cashew nut allergy outcomes.
- The provision of individual participant advice and support by an international board-certified lactation consultant to establish and maintain breastfeeding will enhance breastfeeding rates until at least 6 months postnatal.
- The use of peanuts, peanut butter, cashew nuts and cashew nut spread for the maternal intervention, rather than powders or specific products, will enable rapid low-cost direct translation into practice recommendations.
- This study is single-blinded due to the use of peanuts, peanut butter, cashew nuts and cashew nut spread for the intervention; however, the outcome assessors, statistical analyst and investigators are all blinded to intervention group allocation.

## Introduction

 Overall, peanut allergy prevalence is reported to be between 1% and 2% in the Western nations.^1^ In Australia, 3.1% of infants develop peanut allergy^2^ and 1.5% develop cashew nut allergy.^3^ The Learning Early about Peanut Allergy (LEAP) randomised controlled trial demonstrated that regular inclusion of peanut-containing foods from solid food introduction until 5 years of age, compared with peanut avoidance, reduces the risk of peanut allergy at 5 years of age (1.9% consumption group compared with 13.7% avoidance group, p<0.001).^4^ However, the effect of regular peanut consumption in early childhood was found to be peanut allergen-specific and did not reduce allergic reactions to other foods, such as tree nuts.^5^ Importantly, 76/834 (9.1%) of infants initially screened for the LEAP trial were excluded from participation due to already having peanut sensitisation (peanut skin prick test of >4 mm) prior to dietary peanut exposure,^4^ while another 7 had allergic reactions on their first peanut ingestion at the baseline peanut challenge.^4^ Consistent with these findings, we have demonstrated that some infants have allergic reactions, including anaphylaxis, on first known introduction of egg as early as 4 months of age,^6^ and that these infants have in vitro egg allergen-specific immune responses established at this age.^7^ Furthermore, these immune responses were not altered by early introduction of egg in the infant diet.^7^ Hence, it may be too late for timely allergenic foods introduction in the infant’s diet to change the trajectory for some susceptible infants who are already sensitised/allergic by that age. Thus, intervention strategies are needed earlier in life when the pathways to food allergy development appear to be initiated.

Peanut proteins have been detected in human milk^8^,^11^ and may be important early sources of oral peanut exposure. In animal studies, peanut exposure via maternal milk has been shown to induce oral tolerance.^8^ These tolerogenic effects are assisted by allergen-antibody complexes, which induce allergen-specific T regulatory cells in newborn animals^12 13^ and are also present in human milk.^14^ We have shown that the amount of egg eaten in the maternal diet during lactation influences egg protein concentration in human milk,^15^ and that infant egg-specific antibody blood concentrations are positively associated with maternal egg ingestion during early lactation.^16^ This is consistent with findings that maternal cow’s milk avoidance is associated with lower infant cow’s milk-specific antibody blood concentrations and increased risk of infant cow’s milk allergy.^17^ Hence, we propose that higher maternal consumption of nuts during lactation would increase early infant oral nut exposure in association with tolerogenic nut-specific antibody complexes in human milk.

There is currently insufficient evidence to inform maternal diet recommendations for prevention of food allergy and global allergy prevention guidelines do not provide any advice in this regard. Based on a lack of high-quality evidence, an international expert review concluded that maternal peanut ingestion during breastfeeding, in combination with infant ingestion of peanut, might have a role in peanut allergy prevention, although further studies are required.^18^ We completed a pilot randomised controlled trial^19^ of 109 infants at high risk of developing food allergy (≥2 immediate family members with history of allergic disease). This pilot trial was designed primarily to determine feasibility and compliance with the maternal dietary interventions for the first 6 months of lactation. The pilot trial was not designed with adequate power to determine differences in infant clinical outcomes between the maternal intervention diet groups. In this pilot, we discovered that a maternal high-peanut diet, compared with a low-peanut diet, during the first 6 months of lactation may reduce the risk of infant peanut allergy development. No infants developed peanut allergy after their mothers consumed a high-peanut diet, compared with 8.2% of infants whose mothers followed a low-peanut diet. Consistently, only 2.3% of infants in the maternal high-peanut diet group were sensitised to peanut, compared with 15.2% of infants in the low-peanut group.

Our hypothesis generated from current evidence is that higher maternal peanut and cashew nut consumption during lactation may reduce the development of infant peanut and cashew nut allergies. Thus, we aim to investigate in a randomised controlled trial the effectiveness of higher regular peanut and cashew nut maternal dietary intakes during the first 6 months of lactation as a strategy to prevent peanut and cashew nut allergies in infants.

## Methods and analysis

### Trial design and study setting

This is a multisite, parallel, two-arm (1:1 allocation), single-blinded (outcome assessors, statistical analyst and investigators), randomised controlled trial, known as the Nuts For Babies Study.

### Recruitment

**Figure 1:**
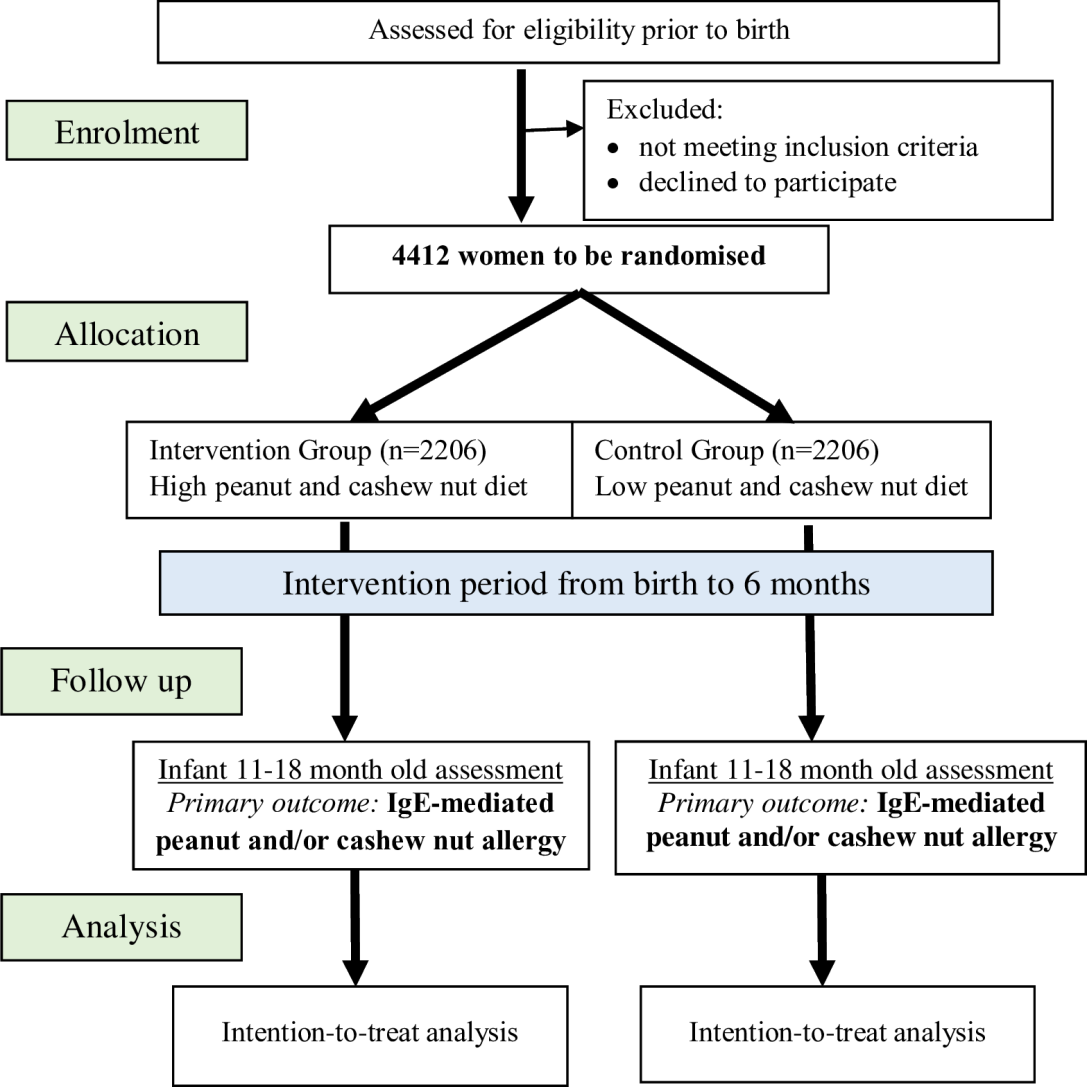
Trial participant flow diagram.

Trial participant flow is illustrated in figure 1. Participant recruitment will occur in the Australian states of Western Australia and Victoria using online (via social media advertising) and in-person (at maternity hospital antenatal clinics/classes) strategies. After screening to ensure inclusion and exclusion criteria are met, a participant information and consent form describing the purpose of the study, the procedures to be followed and the risks and benefits of participation is provided to interested participants. Potential participants are given as much time as they wish to consider participation in the study, have any questions answered or to discuss taking part with their family, friends and/or healthcare professionals. Participants are required to provide written informed consent and are given a copy of their signed consent form. A record of all potential participants screened and their enrolment status is maintained to adhere to the Consolidated Standards of Reporting Trials (CONSORT).^20^ Participants may withdraw their involvement in the trial at any time, without explanation and without prejudice to their future care. Wherever possible, the reason for withdrawal will be recorded. Participants who discontinue or are withdrawn will not be replaced.

### Participant eligibility criteria

The inclusion criteria are women (aged 18–50 years) able to give informed consent, with a singleton pregnancy, who are planning to breastfeed. The exclusion criteria are women with peanut and/or cashew nut allergies, as they would be unable to safely follow the intervention without allergic reactions, and participants who have already participated in this Nuts For Babies Study with a previous child.

### Allocation of interventions

After confirming eligibility, participants provide written informed consent prior to trial randomisation. Trial intervention team research staff use a secure web-based randomisation service embedded within the secure trial-specific Research Electronic Data Capture (REDCap) database. The randomisation service allocates a unique uninformative participant trial identification number and a group allocation according to a computer-generated randomisation schedule, produced by a statistician not otherwise involved in the trial. Randomisation is stratified by state (Western Australia or Victoria), and by any parental/sibling (of the infant) history of eczema/atopic dermatitis or IgE-mediated food allergy,^21^ using randomly permuted blocks of varying sizes. Block sizes will not be disclosed to ensure allocation concealment. Following randomisation, intervention phase research staff explain the participant’s intervention allocation via a telephone call.

### Blinding

Due to the nature of this type of dietary intervention, it is not possible to blind the participants or intervention phase research staff responsible for providing dietary advice and undertaking all participant contact phone calls during the intervention period. However, research staff undertaking the outcome assessments, all investigators and statistical analysts are blinded to intervention allocation. At each contact phone call or appointment, outcome assessors remind participants not to disclose their diet group allocation.

### Interventions

The participants are randomised to either a high peanut and cashew nut diet (intervention) group or a low peanut and cashew nut diet (control) group.

#### High peanut and cashew nut diet (intervention) group

Maternal consumption of at least 60 peanuts and 40 cashew nuts per week from birth until 6 months postnatal.

#### Low peanut and cashew nut diet (control) group

Maternal consumption of no more than 20 peanuts and 12 cashew nuts per week from birth until 6 months postnatal.

The Australian Bureau of Statistics Australian Health survey data found that, on average, Australians consume around 32 g per week of any nuts. This includes peanuts and cashew nuts. Our low nut diet (control) group reflects this with no more than 20 peanuts (approximately 18 g) and no more than 12 cashew nuts (approximately 22 g) per week, thus in total no more than 40 g per week.

Weekly nut ingestion targets can include peanuts and/or peanut butter, and cashew nuts and/or cashew nut spread. To assist with diet compliance a fridge magnet specific to intervention group allocation is provided to each participant including nut conversion details (eg, five peanuts=one teaspoon (5 mL) of peanut butter, four cashew nuts=one teaspoon of cashew nut spread). Participants are encouraged to contact the intervention phase research staff at any stage during the intervention period if they require further dietary adherence assistance.

The dosages of peanut consumption for each group have been matched to those used in the pilot trial,^19^ where 80% compliance was achieved by both allocated groups. The doses in the pilot trial were based on another trial,^16^ where we determined that for each additional egg eaten per week, there was an average 25% increase in egg protein (ovalbumin) breast milk concentration and 22% increase in infant egg-specific IgG4 concentrations (associated with tolerance development). Peanut protein (Ara h 6) has been detected in breast milk samples at similar concentrations to the egg protein (ovalbumin) after 30 g peanuts and one egg ingestion, respectively. Hence, the quantities of peanut chosen for the maternal high-peanut intervention group of at least 60 peanuts eaten per week should induce beneficial infant peanut-specific immune responses in early postnatal life. Maternal consumption of at least 60 peanuts per week in the intervention group is projected to result in a threefold increase in infant peanut-specific IgG4 concentrations above those of the control group (maternal consumption of no more than 20 peanuts per week). The cashew nut quantities are matched for equivalent protein content.

The control group women will be recommended to eat other tree nuts (eg, almonds) as snack suggestions to provide good nutritional sources of energy, protein, fibre, magnesium, B group and E vitamins, as these are also good nutritional sources from peanut and cashew nut consumption. Other tree nuts, such as almonds and hazelnuts, have different nut-specific allergens to those found in peanuts and cashew nuts.

Participants who cease breastfeeding prior to 6 months will remain in the trial and will be asked to continue their allocated peanut and cashew nut diet until their infant is 6 months of age. This is important to continue the consistency of maternalinfant environmental peanut and cashew nut exposure concentrations as would be the usual ‘real-life’ scenario where women would not usually change their diet when they cease breastfeeding. All infants will participate in the follow-up period regardless of duration of breastfeeding or maternal peanut and cashew nut intervention group adherence. Research staff at each site who provide the dietary group allocation advice to participants are trained by the intervention team clinical trial coordinator, and the research staff’s intervention advice is monitored on a 6 monthly basis throughout the trial.

### Intervention compliance

To monitor intervention adherence, every 2 weeks during the intervention period, the participants are sent a mobile phone text with a link to complete a short survey about how much peanut and cashew nut they consumed over the past week, and whether they would like any further advice and support regarding their allocated nut diet. This survey also assesses current breastfeeding status and whether the participants would like to be referred to an international board-certified lactation consultant for any advice and support. For the promotion of breastfeeding, an international board-certified lactation consultant can assist and provide advice to the participants with establishing and maintaining breastfeeding until at least 6 months postnatal.

### Outcomes

The primary outcome for this trial is IgE-mediated peanut and/or cashew nut allergy during infancy. In this trial, infancy is defined as birth until less than 18 months of age.

#### Allergic sensitisation to peanut/cashew nut/hazelnut/almond

The infants have skin prick testing using standard single prick lancets (Entaco distributed by Stallergenes Australia Pty Ltd) on the forearm, to determine allergen sensitisation to peanut, cashew nut, almond and hazelnut, with histamine and control solutions, in accordance with standard clinical methods. All assessment sites use the same commercially available skin prick testing extracts of peanut, cashew nut, almond and hazelnut (Greer Laboratories, USA), positive control histamine (HollisterStier, USA) and negative control 50% glycerin (Greer Laboratories, USA). Sensitisation is defined as a positive skin prick test with mean weal diameter ≥2 mm.

#### IgE-mediated peanut/cashew nut/hazelnut/almond allergy

An infant will be classified as having an IgE-mediated peanut/cashew nut/hazelnut/almond allergy if the infant has had a previous history of anaphylaxis and a positive skin prick test to the same food allergen. The Australasian Society of Clinical Immunology and Allergy (ASCIA) definition of anaphylaxis will be used for this trial: any acute onset illness with typical skin features (urticarial rash or erythema/flushing and/or angioedema), plus involvement of respiratory and/or cardiovascular and/or persistent severe gastrointestinal symptoms; or any acute onset of hypotension or bronchospasm or upper airway obstruction where anaphylaxis is considered possible, even if typical skin features are not present.^22^

**Figure 2:**
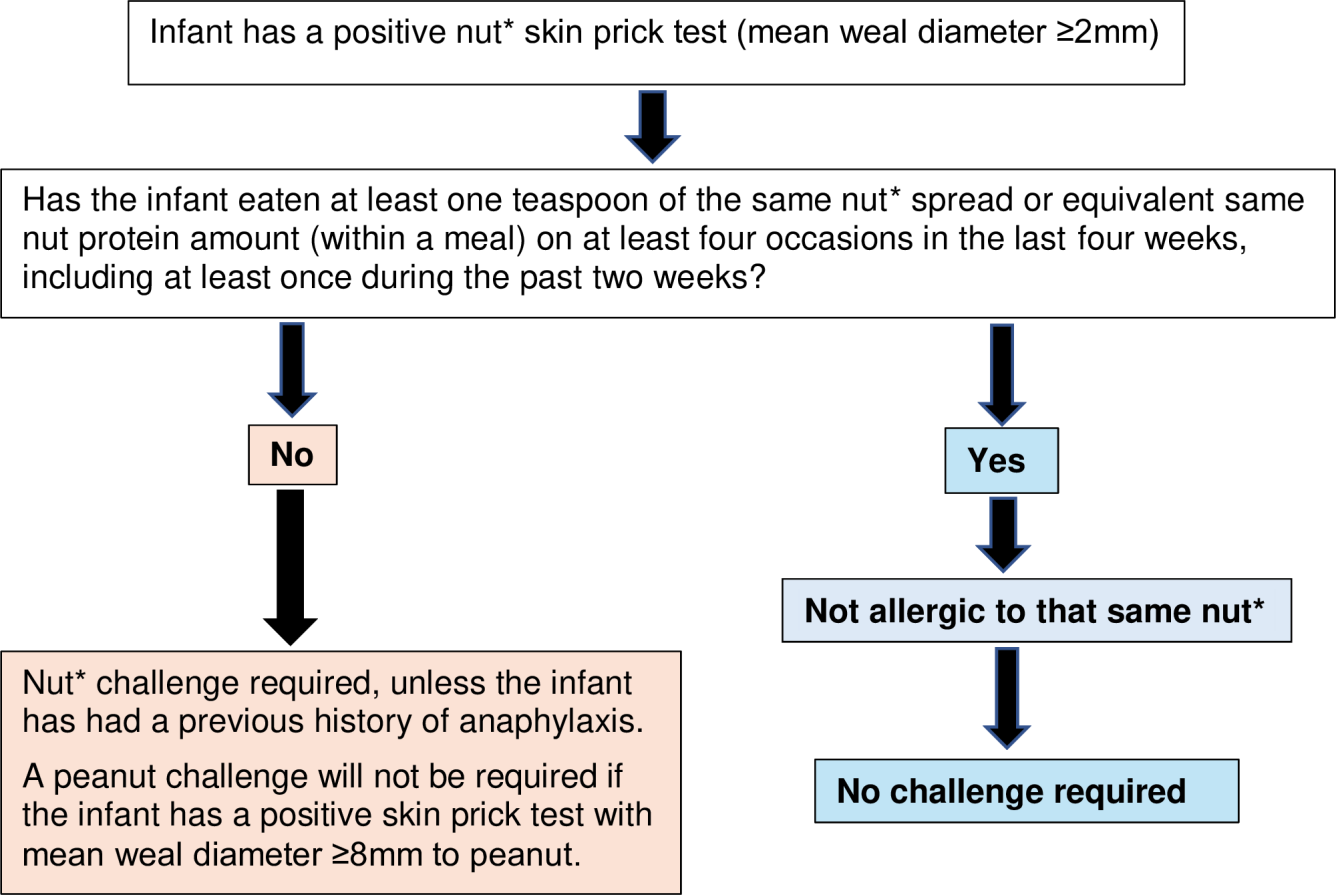
Nuts For Babies Study food challenge decision tree. *Nut=peanut, almond, hazelnut or cashew nut.

Additionally, an infant will be classified as having an IgE-mediated peanut allergy without the need for a peanut food challenge if the infant has a positive skin prick test with mean weal diameter ≥8 mm to peanut.^23^ All other sensitised infants not consuming at least one teaspoon of the same nut spread (within a meal) on at least four occasions in the last 4 weeks, including at least once during the past 2 weeks, will have an in-hospital peanut/hazelnut/almond/cashew nut food challenge. Figure 2 illustrates the trial food challenge decision tree. Peanut/hazelnut/almond/cashew nut challenges with semi-logarithmic incremental doses of smooth peanut butter/hazelnut/almond/cashew nut spread given at 20 min intervals are conducted under medical supervision. Food challenges are considered positive with the occurrence of any moderate or severe dose-limiting symptoms or multiple mild symptoms following standardised scoring and stopping criteria.^24^

#### The secondary outcomes for this trial are:

1. IgE-mediated peanut allergy during infancy.
2. IgE-mediated cashew nut allergy during infancy.
3. IgE-mediated almond allergy during infancy.
4. IgE-mediated hazelnut allergy during infancy.
5. IgE-mediated allergy to peanut and cashew nut during infancy.
6. IgE-mediated allergy to any two (peanut/cashew/almond/hazelnut) nuts during infancy.
7. IgE-mediated allergy to any three (peanut/cashew/almond/hazelnut) nuts during infancy.
8. IgE-mediated allergy to all four (peanut/cashew/almond/hazelnut) nuts during infancy.
9. Allergic sensitisation to peanut during infancy.
10. Allergic sensitisation to cashew nut during infancy.
11. Allergic sensitisation to almond during infancy.
12. Allergic sensitisation to hazelnut during infancy.
13. Allergic sensitisation to peanut and cashew nut during infancy.
14. Allergic sensitisation to any two (peanut/cashew/almond/hazelnut) nuts during infancy.
15. Allergic sensitisation to any three (peanut/cashew/almond/hazelnut) nuts during infancy.
16. Allergic sensitisation to all four (peanut/cashew/almond/hazelnut) nuts during infancy.
17. Medical diagnosis of eczema during infancy. The assessment team nurses will ask whether the infant has had a medical diagnosis of eczema during the 6, 9 and 12-month contacts.

### Participant timeline

**Figure 3:**
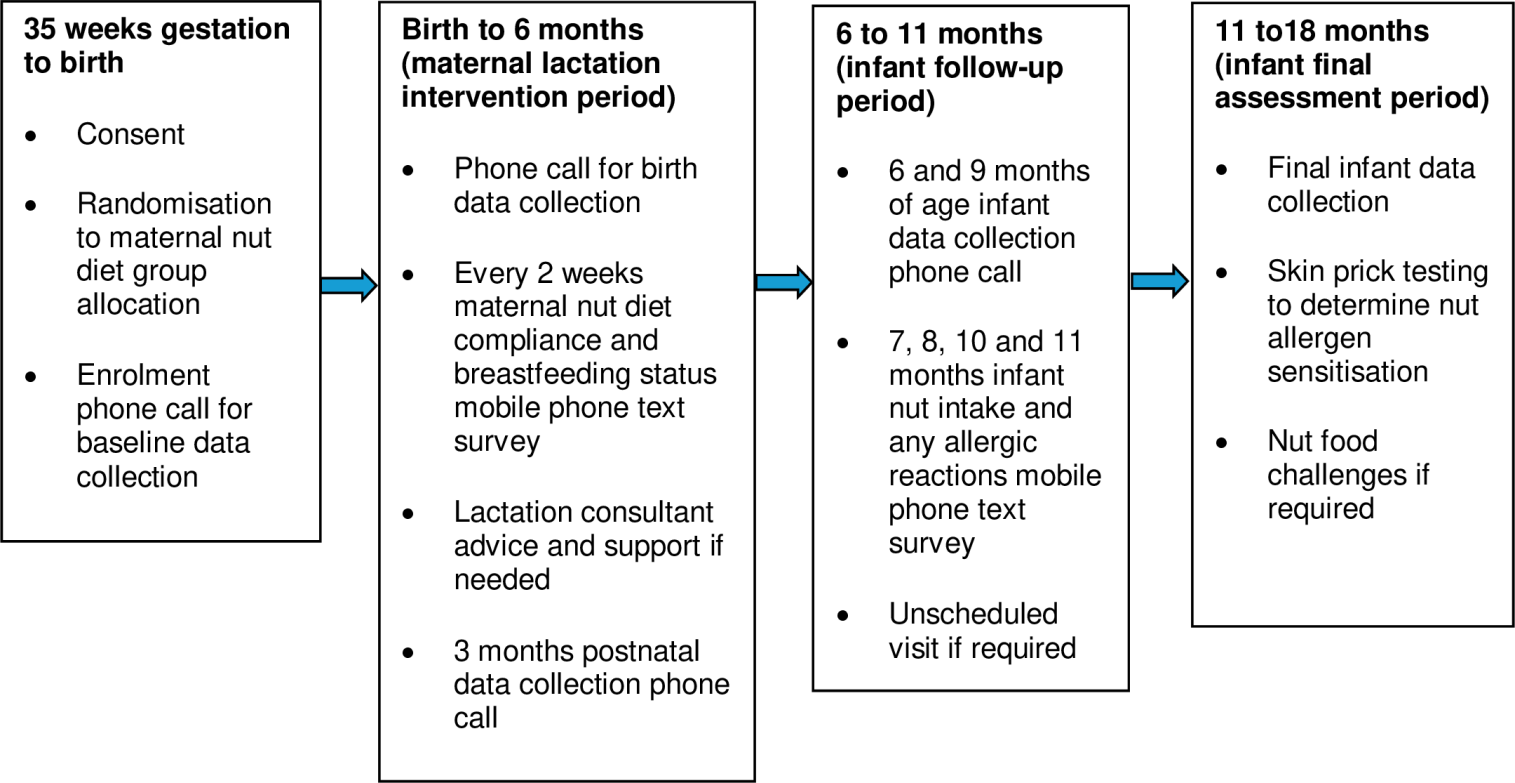
Participants’ (maternal and infant) schedule of enrolment, intervention and assessment activities.

Figure 3 illustrates the participants’ schedule of enrolment, intervention and assessment activities, and table 1 summarises the baseline, intervention and follow-up data collection and assessment time-points.

**Table 1:** Baseline, intervention and follow-up data collection and assessment time-points for the participants and their infants

Data collection	Enrolment phone call: prior to birth	Phone call at 2 weeks of age	Text surveys every 2 weeks: 2 weeks to 6 months	Phone call: 3 months	Phone call: 6 months	Monthly text surveys: 7–11 months	Phone call: 9 months	Infant final assessment: 11–18 months
Baseline data and randomisation	X							
Intervention period		X	X	X	X			
Birth details		X						
Maternal diet compliance		X	X	X	X			
Infant feeding		X	X	X	X	X	X	X
Hospitalisation				X	X		X	X
Follow-up Period					X	X	X	X
Infant eczema				X	X		X	X
Infant peanut and cashew nut spread intake						X	X	X
Nut allergic sensitisation								X
Peanut and/or cashew nut allergy (primary outcome)								X

#### Baseline screening and data collection

Eligibility is confirmed and demographic information collected according to CONSORT guidelines.^20^ For eligible participants, written informed consent is obtained prior to the birth of the infant (from 35 weeks’ gestation onwards).

Baseline data are recorded, including maternal age, education, ethnicity, height, prepregnancy weight, primiparous or multiparous, any infants previously breastfed, parental and/or sibling (of the infant) allergic disease(s) history, any pets, and usual (past month) total household intake of peanuts, peanut butter, cashew nuts and/or cashew nut spread.

#### Two weeks postnatal

Participants are phoned to collect details on infant sex, birth weight, length and head circumference, birth gestation, birth mode, breastfeeding status, any infant formula use, and asked if they have any questions about their assigned nut diet.

#### At 3 months postnatal

Participants are phoned and asked if they have any study-related questions. Information about breastfeeding and any infant formula use is collected. Participants are also asked about any maternal or infant hospital admissions since birth, if their infant has had a medical diagnosis of eczema, and quantities of peanuts, cashew nuts, almonds and hazelnuts (and associated nut spreads) that all members of their household have eaten over the previous month.

#### At 6 and 9 months postnatal

Participants are phoned and asked questions about breastfeeding, solid foods introduction, infant consumption of peanut butter, almond, hazelnut and cashew nut spread, any suspected allergic reactions in infants to peanut/cashew nut/almond/hazelnut, any medical diagnosis of infant eczema, and any maternal or infant hospitalisations. At 6 months (end of intervention period), all participants (both groups) are provided with advice on the introduction of peanut, cashew nut, almond and hazelnut spreads in the infant diet. All families are educated on recognising the signs and symptoms of an allergic reaction and advised what to do in such circumstances.

#### At 7, 8, 10 and 11 months postnatal

Participants are sent a link via mobile phone to complete a short survey to capture age of introduction of peanut butter and cashew nut spread, how much peanut butter and cashew nut spread their infant has eaten over the previous week (once introduced) and to report any suspected allergic reactions to peanut or cashew nut.

#### Final assessment at 11–18 months of age

Participants are asked to attend an appointment at the major paediatric hospital in each state: Perth Children’s Hospital (Perth, Western Australia) and Royal Children’s Hospital (Melbourne, Victoria). If the participant is unable to attend this appointment in-person, assessment research staff will contact them via telephone. The participants are asked questions about breastfeeding, any infant consumption of peanut butter, cashew nut, hazelnut and almond spread, any suspected infant allergic reactions to peanut, cashew nut, hazelnut and almond, and whether their infant has had a medical diagnosis of eczema, use of any eczema treatments, any maternal and/or infant hospitalisations and past month total household consumption of peanuts, cashew nuts, almonds and hazelnuts (and their associated nut spreads). Participating infants have skin prick testing (as described in the Outcomes section), and a food challenge if required (as described in the Outcomes section).

#### Unscheduled visit prior to 11 months of age

If a parent reports that their infant has had an allergic reaction to peanut/cashew nut/almond/hazelnut spread prior to 11 months of age, they will be telephoned by a study doctor. The study doctor will advise either that the parent can continue to offer the nut spread to the infant, or they will be asked to attend for skin prick testing and assessment. At this unscheduled visit, the infant will only be skin prick tested to the nut that their parent has reported their allergic reaction to. The infant will still attend the final assessment between 11 and 18 months of age to undergo skin prick testing to the other nuts. If at the unscheduled visit prior to 11 months of age the infant has a positive skin prick test with mean weal diameter ≥2 mm, the food challenge decision tree (figure 2) will be followed.

### Data collection, management and monitoring

Data are collected by trained research staff at each participating site and entered directly into a trial-specific REDCap database with defined user-level access to facilitate trial management, data collection and blinding as appropriate. A record of all potential participants successfully screened for eligibility and consented is recorded in real time. Once consented and randomised, the REDCap database automatically calculates study milestones for each participant. This information is readily available for research staff to enable scheduling of appointments and phone calls. The trial-specific REDCap database has inbuilt data entry validity checks to ensure immediate resolution of data queries. Data queries are also generated by statisticians during regular blinded reviews of data quality. Electronic data are stored on secure servers with access only granted to authorised study personnel. All data collected are treated with confidence. Data entered by individual study sites are routinely monitored by the clinical trial coordinators to check protocol adherence and study progress. Monthly summary reports are generated, including screening data, enrolment, phone call and appointment completions, and reviewed at trial investigator meetings. Site monitoring to ensure compliance with good clinical practice and the study protocol are conducted at site start-up and then 6 monthly or as required to ensure the integrity of the trial. The trial investigators and The Kids Research Institute Australia (sponsor) will permit trial-related monitoring, audits and regulatory inspections, providing direct access to source data/documents to the approving Human Research Ethics Committee and Institutional Governance review bodies. The trial chief investigator team will review and make protocol amendments if required, monitor overall study progress and make decisions regarding resource allocation at monthly online meetings.

An independent Data and Safety Monitoring Board (DSMB) will safeguard the interests of trial participants by assessing the safety of the interventions during the trial, including individual review of all serious adverse events. The DSMB will also monitor the overall conduct of the trial, including recruitment, compliance, loss to follow-up and withdrawals. A formal charter has been developed to assist with the running of the DSMB, which will consist of a paediatrician, an allergist/immunologist, a lactation consultant and a biostatistician. No interim analyses are planned.

### Sample size

In Australia, during infancy the prevalence of IgE-mediated peanut allergy is 3.1%^2^ and cashew nut allergy is 1.5%,^3^ with 16.7% infants with a peanut allergy also having cashew nut allergy.^3^ Conservatively in this trial, we expect the prevalence of IgE-mediated peanut allergy and/or cashew nut allergy during infancy (primary outcome) to be at least 3.1%. In our pilot trial^19^ using the same peanut maternal intervention/control groups and intervention period, we found no (0%) high-risk infants (due to family history of allergies) developed peanut allergy after their breastfeeding mothers consumed the high-peanut diet, compared with 8.2% infants with peanut allergy after their mothers followed a low-peanut diet (100% relative reduction in risk). Consistently, we also found only 2.3% infants were peanut sensitised in the high-peanut maternal consumption group, compared with 15.2% infants in the maternal low-peanut group (85% relative reduction in risk). Hence, in this trial to be conducted in the general Australian population, not enriched for family history of allergic disease, we expect a minimum reduced effect of 50% on infant peanut and/or cashew nut allergy. Such a reduction in the diagnosis of peanut and/or cashew nut allergy will lead to changes in nut allergy prevention guidelines. To detect a reduction in peanut and/or cashew nut allergy risk from 3.1% to 1.55% (relative reduction of 50%) with 90% power and 95% confidence (alpha-value 0.05), we require 1985 participants per group. To allow for 10% loss to follow-up and withdrawals, including perinatal losses, we will recruit a total of 4412 participants (2206 per group).

### Data analysis plan

Analyses will be performed on an intention-to-treat basis (ie, using a treatment policy strategy for non-compliance within the new estimand framework) according to a pre-specified statistical analysis plan. Data analysts will be fully blinded to group allocation prior to database lock and provided with uninformative treatment codes (eg, A and B) for conducting the analysis. The proportion of infants with IgE-mediated peanut and/or cashew nut allergy will be compared between groups using log binomial regression. Adjustment will be made for variables used to stratify the randomisation and other pre-specified baseline prognostic variables. The difference between groups will be expressed as a relative risk with a 95% CI and two-sided p- value. Log binomial regression models will be used to analyse secondary clinical outcomes. Missing data on the primary and secondary outcomes will be addressed using multiple imputation, implemented separately by treatment group using chained equations. Further details on the multiple imputation approach, including candidate auxiliary variables, will be provided in the statistical analysis plan. In planned subgroup analyses of the primary outcome, we will test for evidence of effect modification by total household peanut and cashew nut dietary intakes. A supplementary per-protocol analysis of the primary outcome will be undertaken in participants who breastfeed to 6 months and adhere to the allocated peanut and cashew nut intakes.

## Ethics and dissemination

### Ethics

Ethical approval has been granted from the Western Australian Child and Adolescent Health Service Human Research Ethics Committee (approval number RGS0000006685), as the lead Human Research Ethics Committee, with Governance site approvals at all participating maternity and children’s hospital sites. The study will be conducted in compliance with the current approved version of the protocol (V.4, 9 December 2024). Any change to the protocol document or informed consent form that affects the scientific intent, study design, patient safety or may affect a participant’s willingness to continue participation in the study will be considered a major amendment and shall have written approval by the lead Human Research Ethics Committee and Governance at each participating site. Participant confidentiality is strictly held in trust by participating investigators and research staff.

### Patient and public involvement statement

This maternal diet intervention food allergy prevention strategy was conceived after being frequently asked by frustrated parents about why their infant had developed a peanut allergy despite following the current guidelines and introducing peanut into their infant’s diet between 6 and 12 months of age. After obtaining the findings from our pilot trial,^19^ we specifically established a Consumer Advisory Group (CAG) to inform the design and provide ongoing input throughout this trial. Our CAG consists of five women who all have lived experiences of breastfeeding and the burden of family allergic disease, having breastfed at least one baby and having family members with allergic disease(s).

During the trial design period, the CAG had input into the type and frequency of participant contacts and data collection time points and highly endorsed the provision of individual lactation consultant advice (if needed) for participants. Prior to the trial commencement, the CAG co-developed the participant information and consent forms, and the recruitment lay language summary and advertising materials. During the conduct of the trial, the CAG will meet three times per year to contribute additional ‘lived experience’ practical suggestions that may assist with trial refinement and to review and make recommendations on any participant feedback comments. Once the trial results are known, our CAG will be involved in the results translation and associated communications.

A qualitative substudy is also being planned to enable participant feedback and input into the practical dietary considerations needed to enhance future translation of the study findings into food allergy prevention recommendations.

### Data sharing

Once the primary trial outcomes are published, the trial data will be available for data sharing. Data sharing requests will need approval by the trial investigator team. Please send requests to Debbie Palmer (debbie.palmer@thekids.org.au). The Australian National Health and Medical Research Council (NHMRC) supports the sharing of outputs from NHMRC funded research including publications and data. All recipients of NHMRC grants must therefore comply with all elements of the NHMRC Open Access Policy (15 January 2018).

### Dissemination

All investigators will be integral in the communication of the trial results. The trial findings will be submitted for peer-reviewed publication and for presentation at appropriate local and international conferences, as well as to the public through various forms of media and public presentations on maternal diet and food allergy prevention. In addition, the trial findings will be disseminated to participants through a one-page lay summary. The Nuts For Babies Study has been designed with the translational plan that outcomes will inform national and international guidelines on maternal diet during lactation and food allergy prevention irrespective of whether the hypothesis is correct.

### Current trial status

The first participant was randomised on 22 July 2024. Recruitment for this Nuts For Babies Study is expected to be completed by December 2027. The final participant primary outcome assessments are expected to be completed by March 2029.
